# IL13RA2 targeted alpha particle therapy against glioblastomas

**DOI:** 10.18632/oncotarget.17792

**Published:** 2017-05-11

**Authors:** Anirudh Sattiraju, Kiran Kumar Solingapuram Sai, Ang Xuan, Darpan N. Pandya, Frankis G. Almaguel, Thaddeus J. Wadas, Denise M. Herpai, Waldemar Debinski, Akiva Mintz

**Affiliations:** ^1^ Department of Radiology, Wake Forest School of Medicine, Winston-Salem, NC 27157, USA; ^2^ Brain Tumor Center of Excellence, Wake Forest University Comprehensive Cancer Center, Winston-Salem, NC 27157, USA; ^3^ Department of Cancer Biology, Wake Forest School of Medicine, Winston-Salem, NC 27157, USA; ^4^ Department of Nuclear Medicine and Radiology, The People's Hospital of Zhengzhou University, Zhengzhou, Henan 450003, China

**Keywords:** glioblastoma, IL13RA2, alpha particle therapy, Actinium-225, Copper-64

## Abstract

Glioblastoma (GBM) is the most aggressive primary malignant brain cancer that invariably results in a dismal prognosis. Chemotherapy and radiotherapy have not been completely effective as standard treatment options for patients due to recurrent disease. We and others have therefore developed molecular strategies to specifically target interleukin 13 receptor alpha 2 (IL13RA2), a GBM restricted receptor expressed abundantly on over 75% of GBM patients. In this work, we evaluated the potential of Pep-1L, a novel IL13RA2 targeted peptide, as a platform to deliver targeted lethal therapies to GBM. To demonstrate GBM-specificity, we radiolabeled Pep-1L with Copper-64 and performed *in vitro* cell binding studies, which demonstrated specific binding that was blocked by unlabeled Pep-1L. Furthermore, we demonstrated real-time GBM localization of [^64^Cu]Pep-1L to orthotopic GBMs using small animal PET imaging. Based on these targeting data, we performed an initial *in vivo* safety and therapeutic study using Pep-1L conjugated to Actinium-225, an alpha particle emitter that has been shown to potently and irreversibly kill targeted cells. We infused [^225^Ac]Pep-1L into orthotopic GBMs using convection-enhanced delivery and found no significant adverse events at injected doses. Furthermore, our initial data also demonstrated significantly greater overall, median and mean survival in treated mice when compared to those in control groups (*p* < 0.05). GBM tissue extracted from mice treated with [^225^Ac]Pep-1L showed double stranded DNA breaks, lower Ki67 expression and greater propidium iodide internalization, indicating anti-GBM therapeutic effects of [^225^Ac]Pep-1L. Based on our results, Pep-1L warrants further investigation as a potential targeted platform to deliver anti-cancer agents.

## INTRODUCTION

Glioblastoma (GBM) is a grade IV primary malignant astrocytoma characterized by extensive angiogenesis, pseudopalisading, hypoxia and infiltrative margins [1]. Chemotherapy and local radiotherapy have not been completely effective due to long term neurological complications and recurrence related mortality [2, 3]. Persistence of invasive cells present in invaded normal brain and therapeutic resistance of GBM stem cells have been identified as causes of GBM recurrence post therapy. These pitfalls of conventional therapy highlight the need for better GBM targeted therapy.

Interluekin-13 receptor alpha 2 (IL13RA2) is a glioblastoma restricted receptor that is abundantly overexpressed in over 75% of GBMs but absent in normal brain tissue, highlighting its potential as a therapeutic target against GBM [4–7]. IL13RA2 was initially postulated to function as a decoy receptor to functionally sequester interleukin 13 (IL13) and prevent it from binding to the heterodimer IL13RA1/IL4RA receptor (abundantly found within normal brain and tumors), which binds to both IL13 and IL4 [5]. IL13RA2 could therefore prevent apoptosis that would otherwise be initiated by IL13 binding to physiologically abundant receptor on GBM through the STAT6 pathway but its specific role is yet to be clearly elucidated and is an active area of investigation. However, recent evidence has demonstrated a potential role of IL13RA2 in activating the Scr/PI3K/Akt/mTOR pathway [8]. IL13RA2 is internalized upon binding to IL13 and its derivatives in GBM cells, making it an attractive target for cytotoxic therapeutics [9]. In addition, a recent report from a Phase I clinical study has described significant regression of a recurrent GBM in a patient after intracavitary and intraventricular infusion of chimeric antigen receptor (CAR) T-cells targeting IL13RA2 expressing GBM cells, further highlighting the therapeutic advantage of targeting IL13RA2 expressing tumor cells [10, 11].

Peptide ligands can be designed to function as molecular scaffolds specifically targeting IL13RA2 expressing GBM cells with molecular therapeutics while sparing normal brain tissue [6, 7, 12, 13]. Using peptides conjugated to lethal irradiation may be advantageous compared to using larger proteins and antibodies due to their small size and resulting relatively brisk plasma clearance. Pandya *et al*. discovered an IL13RA2 targeting peptide, *peptide-1 linear* (Pep-1L) that showed promising specificity *in vitro* and *in vivo*. Specifically, they evaluated GBM localization of intravenously injected cy5.5 conjugated Pep-1L in mice and found the peptide accumulation within GBM tissue up to 288 hours post injection [14]. Furthermore, Solingapuram *et al*. have demonstrated IL13RA2 specific binding of [^64^Cu]Pep-1L after systemic injection in peripheral mouse tumor models [15]. These results highlighted the potential of using Pep-1L as a molecular scaffold to deliver anti-GBM therapy. In this work, we therefore aim to validate Pep-1L targeting after administration via convection enhanced delivery (CED) using real-time PET/CT for the first-time. Furthermore, we explore using Pep-1L conjugated to cytotoxic α-particle emitting radioisotopes against GBM. α-particle emitters are attractive because they emit short ranged and powerful particles which could be targeted to GBM cells via tumor-specific biomarkers [16, 17]. Actinium-225 (Ac-225) is an α-particle emitter with a 10-day half-life that has been shown to be effective at causing tumor cytotoxicity when targeted to tumor specific biomarkers in various different cancers. ^225^Ac releases 4 alpha particles upon decay, which have a short range of only 50–100 μm but a high linear energy transfer (~80 keV/μm). α-particles cause irreparable double strand DNA breaks only within targeted cells but not in surrounding normal tissue, resulting in targeted GBM cell killing at very low doses [18].

## RESULTS

### Radiolabeling Pep-1L with Cu-64 and Ac-225

**Figure 1:**
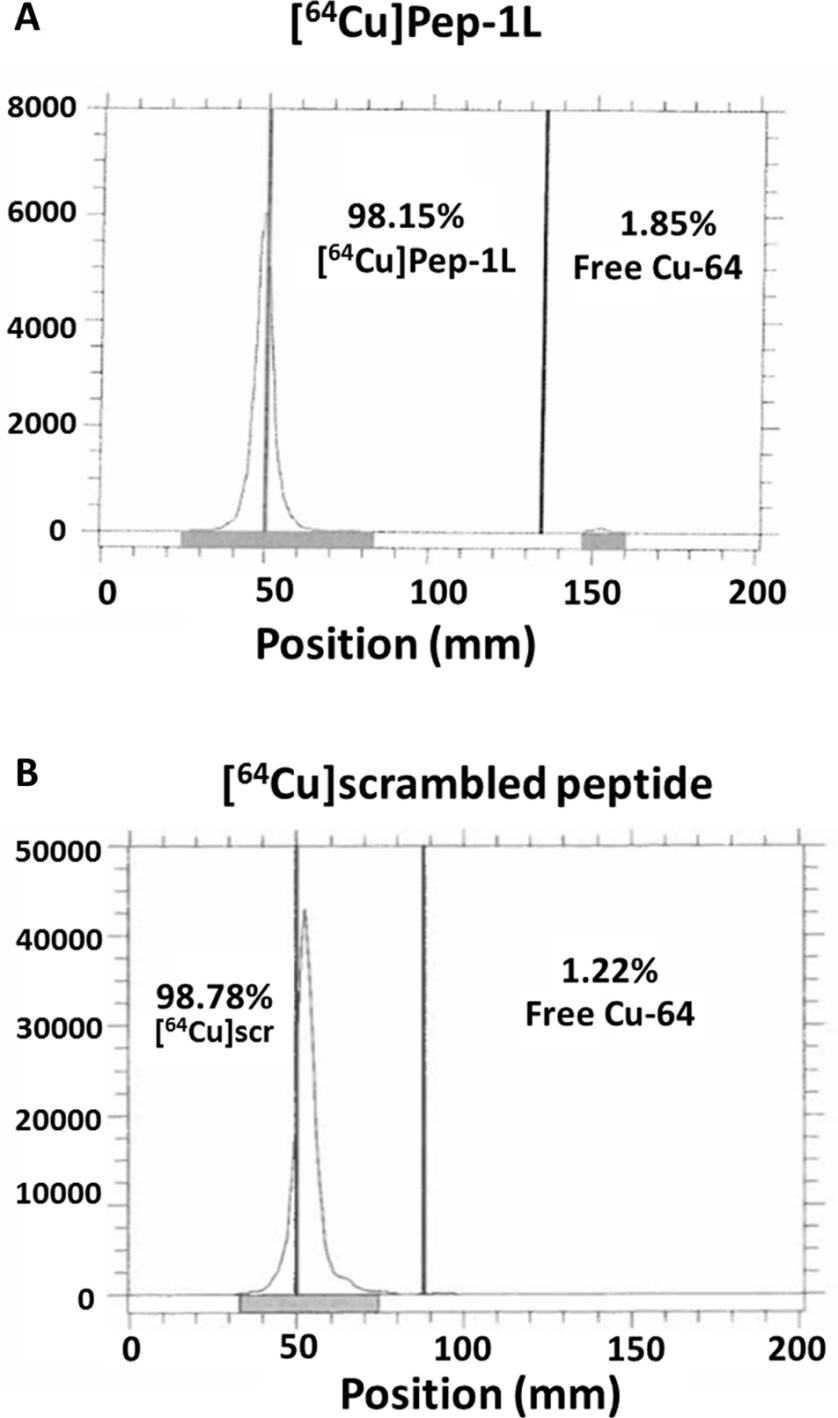
Radiolabeling Pep-1L and scrambled peptide to Cu-64 Radio-TLC analysis showed effective radiolabeling of Pep-1L (**A**) and scrambled peptide (scr) (**B**) to Cu-64 in 0.1M NH4OAc buffer (pH 5.5) at 75°C. Both peptide analogues showed > 98% radiochemical purity.

NOTA-chelated peptides (both Pep-1L and scrambled peptides) were radiolabeled to copper-64 (Cu-64) in 0.1 M NH_4_OAc buffer (pH 5.5) at 75°C for 1 hour using standard methods [15]. Radiolabeled peptide, [^64^Cu]Pep-1L showed > 98% radiochemical purity (Figure 1). Similarly, DOTA chelated peptide was radiolabeled to Ac-225 using previously reported methods [15]. [^225^Ac]Pep-1L showed > 95% radiochemical purity.

### [^64^Cu]Pep-1L shows specificity towards IL13RA2 expressing patient derived xenograft cells *in vitro*

**Figure 2:**
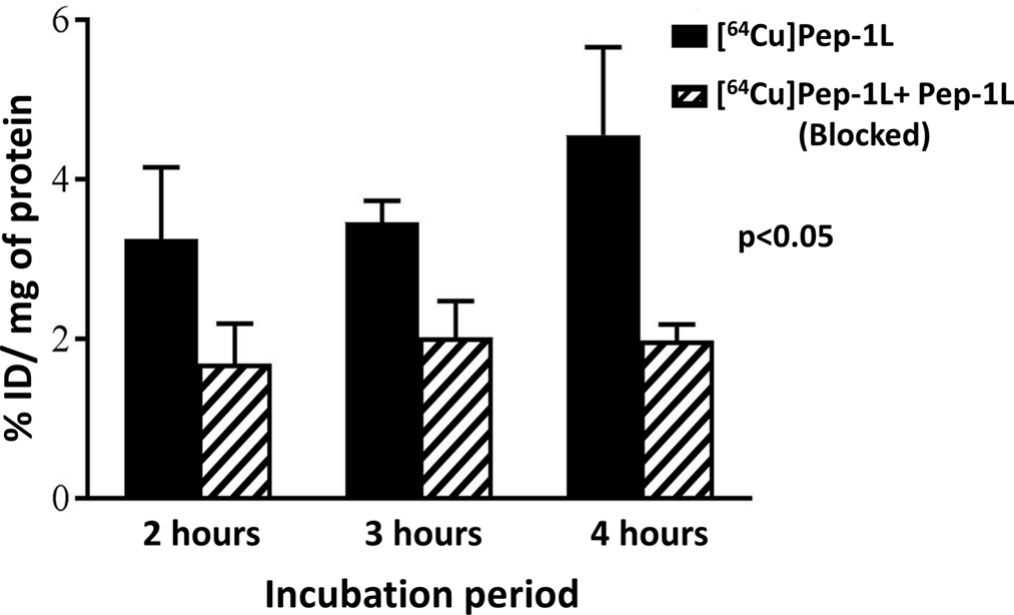
*In vitro* specificity of [^64^Cu]Pep-1L towards IL13RA2 expressing PDX cell line [^64^Cu]Pep-1L showed greater binding to IL13RA2 expressing PDX cells in comparison to its binding to PDX cells blocked with unlabeled Pep-1L at 2 hour, 3 hour and 4 hour incubation periods. This shows IL13RA2 specific cell binding of [^64^Cu]Pep-1L. Data represented as means +/− SEM.

*In vitro* cell uptake assay was performed on a patient derived human glioblastoma cell line (PDX) with and without unlabeled blocking peptide to confirm the specificity of [^64^Cu]Pep-1L. We observed uptake of [^64^Cu]Pep-1L in the IL13RA2 expressing PDX cell line (3–5% injected dose (I.D.)/mg of protein), which was inhibited by blockade using unlabeled Pep-1L after 2 hour, 3 hour and 4 hour incubation periods (Figure 2). These results demonstrate specific binding of Pep-1L to IL13RA2.

### [^64^Cu]Pep-1L delivered via CED demonstrates localization within orthotopic GBMs

**Figure 3:**
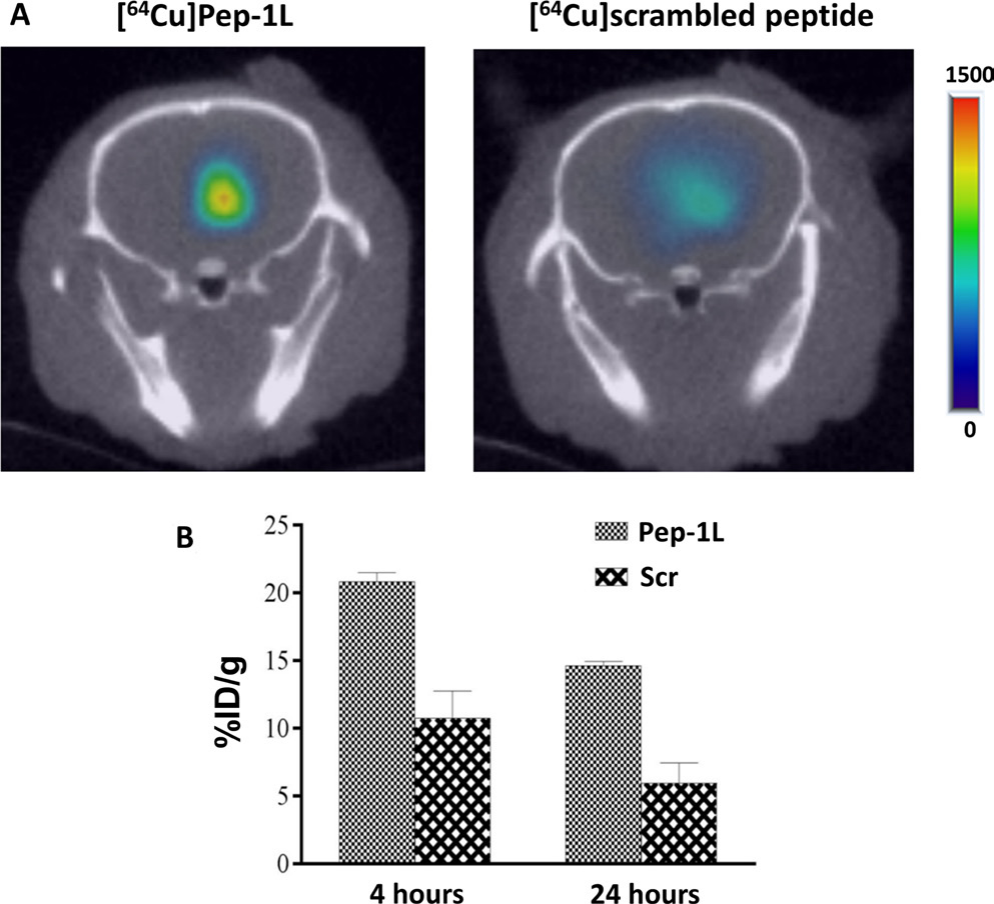
CED of [^64^Cu]Pep-1L shows IL13RA2 specific localization within U251 GBMs *in vivo* (**A**) Athymic mice were intracranially injected with U251 human GBM cells and tumor establishment was tracked using *in vivo* bioluminescent imaging. Upon establishment of GBMs, [^64^Cu]Pep-1L (~150-180 μCi (6.6MBq)/8 μL) and ^64^Cu-scrambled peptide (~150-180 μCi (6.6MBq)/8 μL) were intracranially infused. MicroPET/CT imaging revealed GBM tissue specific localization of [^64^Cu]Pep-1L, delivered via CED in mice bearing orthotopic U251 GBMs. MicroPET/CT imaging also revealed non-specific, dispersed distribution of similarly delivered [^64^Cu]scrambled peptide in mice bearing U251 GBMs. (**B**) Post-necropsy biodistribution analysis showed ~2 fold greater localization of [^64^Cu]Pep-1L within brains of mice when compared to [^64^Cu]scrambled peptide. Data represented as means +/− SEM.

MicroPET/CT imaging studies were performed 4 hours and 24 hours post intracranial infusion of [^64^Cu]Pep-1L and untargeted control ([^64^Cu]scrambled peptide). ROI analysis of microPET/CT imaging data showed focal and increased [^64^Cu]Pep-1L accumulation in the brain of mice, which was ~2-fold greater compared to untargeted control [^64^Cu]scrambled peptide that showed a more dispersed distribution within brain (Figure 3A). To confirm this tumor targeting, we performed post-necropsy analysis on the mouse brains and found that mice that received [^64^Cu]Pep-1L showed significantly higher radioactive uptake within the brain when compared to mice that received untargeted control [^64^Cu]scrambled peptide (Figure 3B). These results from the post-necropsy biodistribution study therefore validate the microPET/CT imaging data and confirm GBM targeting of Pep-1L after CED.

### Safety and initial therapeutic effect of [^225^Ac]Pep-1L against orthotopic IL13RA2-expressing GBM tumors

**Figure 4:**
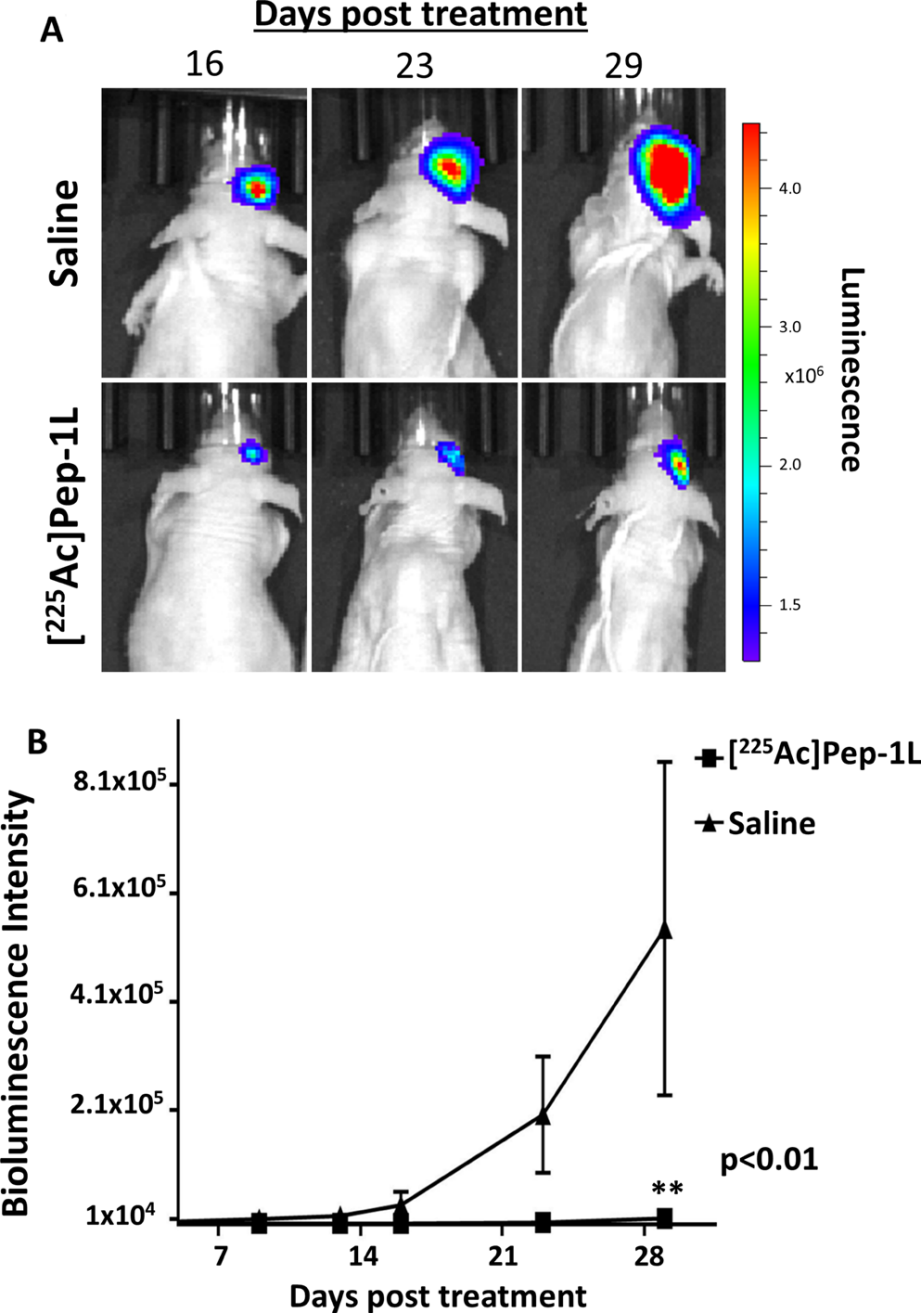
[^225^Ac]Pep-1L delays tumor progression, reduced tumor volume and tumor cell viability *in vivo* against GBMs *In vivo* bioluminescent imaging of mice bearing established *fLuc*-expressing U251 GBMs. (**A**) Mice infused with [^225^Ac]Pep-1L through CED (*n* = 8) showed reduced bioluminescence signal when compared to mice in control groups (*n* = 7) over a period of 29 days post infusion indicating delayed progression of established GBMs and reduced tumor cell viability upon therapy. (**B**) Quantification of bioluminescent signal showed that mice infused with [^225^Ac]Pep-1L had reduced tumor volumes when compared to mice in control groups. Data represented as means +/− SEM. Student's *t*-test showed significant difference between both the experimental groups (*p* < 0.01).

**Figure 5:**
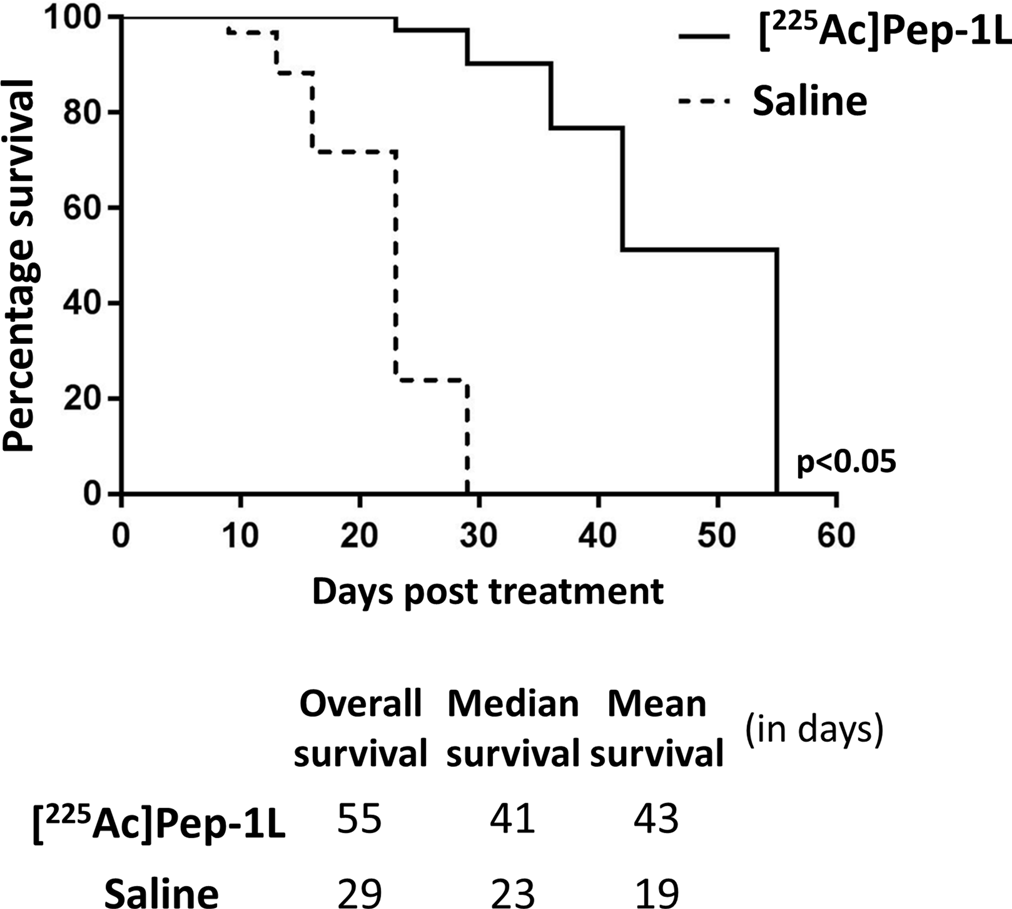
CED of [^225^Ac]Pep-1L *in vivo* shows significantly greater efficacy compared to control groups Kaplan-Meier curve showing significantly greater overall survival, median survival and mean survival (*p* < 0.05) of mice treated with [^225^Ac]Pep-1L when compared to mice in control group. Efficacy of [^225^Ac]Pep-1L against orthotopic GBMs when infused via CED is demonstrated by the greater overall survival, median survival and mean survival.

To examine the safety of [^225^Ac]Pep-1L delivered via CED on orthotopic GBMs, we radiolabeled Pep-1L with Ac-225 using standard methods [19]. 1 μCi (0.04MBq) of [^225^Ac]Pep-1L was infused into orthotopic GBMs using CED (*n* = 8). We did not observe any clinical toxicity due to the injected dose. In addition to monitoring safety, we also gathered preliminary anti-tumor activity of [^225^Ac]Pep-1L by monitoring tumor growth with regular *in vivo* bioluminescent imaging, as U251 GBM cells were modified to express firefly luciferase (*f*Luc). We found that mice treated with [^225^Ac]Pep-1L showed delayed tumor progression (Figure 4A). Quantification of bioluminescent signal showed that mice treated with [^225^Ac]Pep-1L had reduced tumor volume when compared to the mice in control group as indicated by the reduction in tumor bioluminescent signal (Figure 4B) (*p* < 0.01). Furthermore, mice treated with [^225^Ac]Pep-1L showed decreased weight loss when compared to mice in control group, indicating increased intracranial tumor burden in control animals. Furthermore, mice treated with [^225^Ac]Pep-1L demonstrated significantly increased survival, with an overall survival of 55 days, median survival of 41 days and mean survival of 43 days post [^225^Ac]Pep-1L administration compared to control group that demonstrated an overall survival of 29 days, median survival of 23 days and mean survival of 19 days (*p* < 0.05) (Figure 5).

### [^225^Ac]Pep-1L causes double strand DNA breaks, GBM cytotoxicity and reduces cell proliferation in orthotopic U251 GBMs

**Figure 6:**
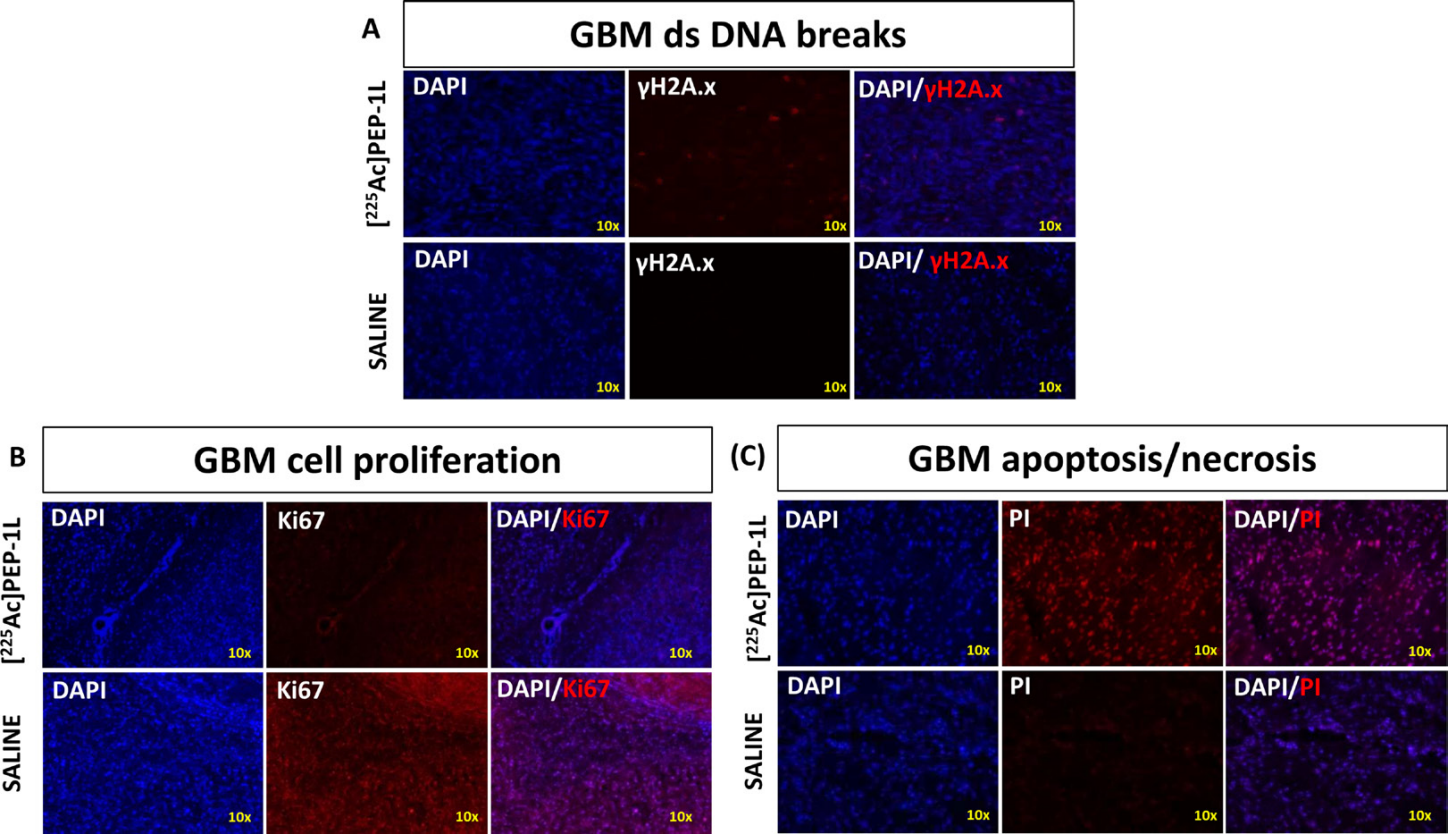
[^225^Ac]Pep-1L causes double strand DNA breaks, GBM cytotoxicity and reduces cell proliferation in orthotopic U251 GBMs Histological analysis of GBMs from mice treated with [^225^Ac]Pep-1L showed (**A**) increased γH2A.X sub-nuclear accumulation indicating fatal double strand DNA breaks, (**B**) lower expression of Ki67 indicating reduced tumor proliferation and progression, thereby supporting bioluminescent data and (**C**) greater internalization of propidium iodide indicating tissue necrosis/apoptosis, likely to be a result of damage by alpha particles when compared to GBMs of mice in control group.

To visualize molecular markers of α-particle induced cytotoxicity, immunohistological analysis was performed on the brains of mice from both groups. GBMs in mice treated with [^225^Ac]Pep-1L showed significantly greater sub-nuclear accumulation of γH2A.X which indicates lethal double strand DNA breaks caused by ionizing effect of α-particles when compared to GBMs of mice from control group (Figure 6A) [20]. We also observed lower Ki67 expression in GBMs of mice treated with [^225^Ac]Pep-1L when compared to GBMs of mice in control group (Figure 6B) indicating reduced tumor proliferation, in line with our *in vivo* bioluminescent imaging data. In addition, we also observed greater propidium iodide internalization in GBMs of mice treated with [^225^Ac]Pep-1L when compared to GBMs of mice in control groups (Figure 6C), indicating potential tumor tissue necrosis/apoptosis resulting from lethal DNA damage. These initial results corroborate the therapeutic response of our [^225^Ac]Pep-1L on orthotopic GBMs.

## DISCUSSION

Targeting lethal radioisotopes to cancer, known as radioimmunotherapy, has had limited success against solid tumors with the exception of thyroid cancer, which naturally takes up untargeted radioactive iodine isotopes via the sodium-iodine symporter. There are many factors that have prevented widespread successful deployment of targeted radiotherapy that are now being overcome and have sparked a renaissance in such therapeutic strategies. Past shortcomings include the pharmacokinetics of the ligand, the strength of the radioisotope and delivery method. Initial therapeutic applications of radioimmunotherapy generally involved conjugating β-emitting isotopes, like iodine-131, to whole antibodies that target a tumor biomarker. However, one challenge faced by this paradigm is that the radioactive antibody remained in the blood for an extended period and exposed normal organs, which limited the dose of radioactivity administered. However, many researchers have now significantly decreased the size of their ligands by deploying antibody fragments, peptides or small molecules, and consequently increasing the tumor-to-background ratio [21]. One common approach uses peptides as a platform to deliver toxic therapeutics such as radioisotopes. Therefore, in this work we chose to utilize Pep-1L to target IL13RA2, as a therapeutic delivery platform against GBM. We and others have demonstrated that IL13RA2 is an attractive tumor-restricted biomarker that is highly over-expressed on the vast majority of GBMs and is internalized after ligand engagement. Importantly, we have shown that IL13RA2 is overexpressed on GBM stem cells, which have been reported to overexpress drug efflux pumps and are relatively insensitive to conventional radiation therapy [4, 7]. These characteristics as well as lack of significant expression of IL13RA2 on normal tissues have instigated the development of a number of therapeutics against IL13RA2 [6, 12, 22–24].

However, additional challenges remain in delivering toxic payloads to GBM. Recurrent disease is a major cause of GBM patient mortality and is thought to occur from persistence of therapy resistant infiltrative cancer cells [25–27]. Furthermore, systemically administered therapeutics generally result in ineffective concentrations of drugs in tumor regions and normal brain containing infiltrative GBM cells [28–30]. Therefore, we and others are exploring novel methods of delivering therapies to GBM. One such delivery modality is Convection Enhanced Delivery (CED), where drug is delivered under constant pressure, enabling it to penetrate deeply into surrounding tumor and brain tissue that may contain infiltrative cells [31–33]. However, a significant cause of failure using this approach in early CED trials has been the lack of real-time imaging to verify proper tumor coverage [34]. For example, in an earlier CED trial using IL13-based cytotoxin, it was later estimated that only about 20% of the tumor volume was covered by the therapy [34], which greatly contributed to only showing modest benefits and not meeting the trial endpoints. While the CED method has drastically improved since the early trials by using innovative catheters that prevent backflow, it has become standard practice to use some type of real-time imaging to ensure proper catheter placement and tumor coverage. We therefore aimed to develop a Pep-1L specific probe that would (a) validate Pep-1L as a platform to deliver therapeutics using CED; and (b) develop a real-time specific probe that would predict tumor targeting as well as quantitate the dose of therapy expected to reach the tumor based on biomarker expression, a feat not accomplished by the current generic imaging surrogates like gadolinium that are used in conventional CED. Therefore, one focus was to demonstrate our ability to molecular target GBM with a novel IL13RA2-targeted peptide after CED using PET/CT.

To demonstrate tumor specific delivery of Pep1L, a novel peptide discovered to target IL13RA2, we radiolabeled it with positron emitting Cu-64 and demonstrated its ability *in vitro* and *in vivo* to target IL13RA2-expressing human GBMs. Importantly, when intratumorally infused into mice bearing orthotopic human GBMs, IL13RA2-targeted [^64^Cu]Pep-1L showed GBM-specific localization compared to similarly infused [^64^Cu]scrambled peptide. We also witnessed increased focal retention of the intratumorally infused [^64^Cu]Pep-1L when compared to [^64^Cu]scrambled peptide 4 hours and 24 hours post intracranial infusion. Given this successful targeting of orthotopic tumors *in vivo*, we initiated a pilot study aiming to show the safety of Pep-1L radiolabeled to Ac-225, a potent α-particle emitter that has shown promise in early clinical trials [17, 35, 36]. In addition to optimized ligands, using more powerful α-emitting isotopes has propelled the field of radioimmunotherapy forward. α-particles are estimated to be 500–1000x more powerful than β-emitters, but have the added benefit of an ultra-short killing range, making it more safe to the surrounding organs [37]. This is especially beneficial for treating diseases such as GBM, where infiltrating tumor cells exist in significant number in normal brain tissue. Additionally, GBMs are often characterized by pseudopalisades, which are areas of hypoxia where residing GBM cells are relatively resistant to γ and β irradiation due to lack of reactive oxygen species (ROS) [38–40]. The use of α-particles avoids this potential issue as their tumor cytotoxicity is the result of irreparable double stranded DNA breaks and does not rely on the presence of ROS [18]. Therefore, we performed initial proof-of-concept safety studies by infusing 1μCi (0.04MBq) of [^225^Ac]Pep-1L into orthotopic human GBM xenografts using CED and observed no adverse events, likely secondary to the demonstrated targeting of Pep-1L as well as the use of ultra-short range α-particles. Furthermore, in addition to safety, these initial pilot experiments demonstrated significant reduction in tumor volume, reduction in tumor cell viability and increased survival when compared to control group. These initial results validate the further study of Pep-1L as a viable delivery platform for anti-tumor therapeutics delivered via CED against GBM.

There are numerous clinical strategies utilizing radioimmunotherapy that are beginning to show positive results in clinical trials and practice. For example, Radium-223 was approved by the FDA against prostate cancer bone metastasis as the first agent to extend survival in patients with castrate resistant prostate cancer with bone metastasis [37]. This agent targets osteoblastic activity characteristically found in the vicinity of prostate cancer bone metastasis [37, 41, 42]. Astatine-211 labeled anti-tenascin targeted therapy was evaluated in a phase-1 trial in primary and metastatic brain tumor patients [43]. Therapeutic benefit of Ac-225 labeled anti-CD33 monoclonal antibody was evaluated in a phase-1 clinical trial in acute myeloid leukemia (AML) patients where median progression free survival (PFS) and median overall survival of 2.7 months and 5.5 months respectively were observed [44, 45]. Given our initial promising results in our current experiment, further evaluation of [^225^Ac]Pep-1L delivered via CED is ongoing.

## MATERIALS AND METHODS

### Animals

Male athymic nude mice and rats were purchased from Taconic Farms (Germantown, NY). All colonies were housed in a pathogen-free facility of the Animal Research Program at Wake Forest School of Medicine under a 12:12-h light/dark cycle and fed ad libitum. All animal experiments were conducted under IACUC approved protocols in compliance with the guidelines for the care and use of research animals established by Wake Forest Medical School Animal Studies Committee.

### Cell culture

U251 human glioblastoma cell line genetically engineered to express firefly luciferin (*f*Luc) was cultured in DMEM- high glucose media (Corning, NY) supplemented with 10% FBS (Invitrogen, Carlsbad, CA) and 1% Pen-Strep (Gibco, Grand Island, New York). PDX cell line was cultured in serum free neurobasal media (ThermoFisher Scientific, Somerset, NJ) supplemented with 1% anti-anti (ThermoFisher Scientific, Somerset, NJ), 50 μg human epithelial growth factor (hEFG) and 50 μg human fibroblast growth factor beta (hFGF-β).

### Radiolabeling of Pep-1L Cu-64 to and Ac-225

Ac-225 was purchased from Oak Ridge National Laboratory. For Ac-225 labeling, the prepared DOTA-Pep-1L (CPC-scientific, San Jose, CA) was incubated with Ac-225 at 70^°^C for 50 minutes. The TLC plates were scanned on a BioScan Imaging Scanner. Cu-64 was purchased from Washington University in St. Louis. The custom peptide specific to IL13RA2 and a scrambled peptide were conjugated with NOTA by CPC scientific Inc (San Jose, CA). Both the peptides, Pep-1L and scrambled peptide-NOTA were radiolabeled with Cu-64 according to the previously reported methods [15]. Briefly, 20 μg of NOTA-peptide was dissolved in 0.1 M ammoniumacetate (NH_4_OAc) aqueous buffer solution pH 5.5. [^64^Cu]Cl_2_ was converted to [^64^Cu](OAc)_2_ by dissolving in 100 μL of 0.1M NH_4_OAc pH 5.5. The resultant [^64^Cu](OAc)_2_ ~1-2 mCi (0.04-0.08MBq) was then added to the conjugated NOTA-peptide aliquot in an Eppendorf tube and heated at 75^°^C for 1 hour. Radiochemical purity was determined by radio-TLC (MK-C18 reversed phased TLC plates). Around ~1 μL of the reaction mixture was applied on the C18-reversed phase TLC plate and developed with 10% NH_4_OAc: MeOH (30:70) as the mobile phase. After the completion of reaction (through TLC analysis), the reaction mixture was quenched with 5 mM EDTA aqueous solution and stirred for an additional 15 minutes at 30°C. The final product was passed through 0.22 μm filter and re-analyzed by iTLC strips with 5 mM EDTA solution as mobile phase. The TLC plates were scanned on a BioScan Imaging Scanner.

### Cell uptake assay

The *in vitro* specificity of [^64^Cu]Pep-1L towards IL13RA2 was confirmed using human patient derived glioblastoma cell line (PDX) acquired from Mayo Clinic (Rochester, MN) following previously reported methods. PDX cells were harvested using trypsin (ThermoFisher Scientific, Somerset, NJ). Cells were counted using a hemocytometer and 1 × 10^5^ PDX cells were then seeded into each well of a 6-well culture plate coated with 0.02mg/mL laminin (ThermoFisher Scientific, Somerset, NJ). PDX cells were incubated overnight at 37°C and 5% CO_2_ in an incubator. On the day of experiment, fresh solution of Pep-1L was made at a concentration of 5μM in serum free neurobasal media and was used as the blocker solution. The blocker solution was added 15 minutes prior to addition of radiotracer. PDX cells were incubated with [^64^Cu]Pep-1L (0.5 μCi (0.02MBq) /well) for 2 hour, 3 hour and 4 hour incubation periods (*n* = 3) at 37°C. Residual fluid was removed by pipette, and 200 μL of 0.1% aqueous sodium dodecylsulfate lysis buffer solution was added to each well. The radioactivity was counted using the Wallac 1480 Wizard gamma counter (Perkin Elmer, Turku, Finland). Additional 20 μL aliquots were taken in triplicate from each well for protein concentration determination. The uptake data in each sample from each well and the standard counts for each condition were expressed as counts per minute (cpm) of activity and are decay corrected for elapsed time. The cpm values of each well were normalized to the amount of radioactivity added to each well and the protein concentration in the well and expressed as percent uptake relative to the control condition. The data were expressed as %ID/mg of protein present in each well with *p* values ≤ 0.05 considered statistically significant.

### Stereotactic intracranial injections

Orthotropic glioblastomas were implanted by stereotaxic implantation of 10^5^ actively growing U251 glioma cells in nude mice. Briefly, mice were weighed and anesthetized with a mixture of 114 mg/kg Ketamine and 17 mg/kg xylazine. Mice were placed in a stereotaxic setup and a hole was made 2 mm lateral and 0.5 posterior to the bregma in the right cerebral hemisphere through a scalp incision. Stereotaxic injection was performed using a Just for Mice stereotaxic apparatus (Harvard Apparatus, Holliston, MA), with injection of 10-μL Hamilton syringe (Sigma-Aldrich, St. Louis, MO) through the hole to a depth of 3.2 mm. A Nanomite programmable syringe pump (Harvard Apparatus, Holliston, MA) delivered constant infusion of U251 cells (10^5^/5 μL), [^64^Cu]Pep-1L (~150-180 μCi (6.6MBq)/8 μL), ^64^Cu-scrambled peptide (~150-180 μCi (6.6MBq)/8 μL) and [^225^Ac]Pep-1L (1 μCi (0.037MBq)/5 μL) at a rate of 1 μL/min. All mice received an analgesic (Ketaprofen) and were monitored for body weight, ambulatory, feeding and grooming activities.

### Small animal microPET/CT imaging

Mice bearing orthotopic U251 GBMs were placed in an induction chamber containing 2% isoflurane/oxygen and then secured to a custom double bed; anesthesia was maintained via nose-cone at 1% isoflurane/oxygen for the dynamic imaging procedure. The mice were scanned for 20 minutes using Triumph PET/CT scanner (TriFoil, Chatsworth, CA) 4 hours and 24 hours post infusion. The regions of interest (ROIs) were generated for both experiment groups.

### Post-necropsy biodistribution analysis

After the completion of micro PET data acquisition, mice were euthanized to measure radioactive uptake within brains of mice. Brains from mice infused with [^64^Cu]Pep-1L and [^64^Cu]scrambled peptide were extracted, weighed and then counted on a Beckman Gamma 8000 well counter (Beckman Instruments, Inc., Irvine, CA) with a standard dilution of the injectate. The percentage of the injected dose per gram of tissue (%ID/g) was calculated.

### Bioluminescent imaging

Mice were anaesthetized using 2% mixture of isoflurane and oxygen in an induction chamber. Each mouse was intraperitoneally injected with 10 ml/g of D-luciferin (150 μg/mL) and placed in an *in vivo* IVIS bioluminescent imaging system (PerkinElmer, Waltham, MA) that supplied mice with 2% mixture of isoflurane and oxygen through nose cones. Regions of interest were drawn around bioluminescent signals in the resulting pictures. After bioluminescent imaging, mice were returned back to their respective cages upon regaining consciousness.

### Extraction and cryopreservation of rodent brains

Mice were anesthetized; transcardially perfused with PBS followed by 4% PFA. Brains were removed; postfixed in 4% PFA, cryoprotected in 30% sucrose, covered with tissue embedding medium, snap frozen in liquid nitrogen and stored at −80°C.

### Tissue sectioning

Serial, 20 μm thick, transverse sections of the frozen tissues were obtained using a cryostat (Microm HM 500, Zeiss, Germany) at −20°C and were mounted on Super Frost Plus microscope slides (ThermoFisher Scientific, Somerset, NJ) in series of six and were stored at −20°C.

### Immunohistochemistry

Sections were dried at room temperature for an hour, rehydrated in PBS, permeabilized with 0.5% Triton X-100 (Sigma) in PBS solution and blocked to saturate non-specific antigen sites using 5% (vol/vol) goat serum-PBS (Jackson Immunoresearch Labs, West Grove, PA) at 4°C overnight. On the next day, the sections were incubated with 0.05% Tween 20 and incubated in anti-Ki67 antibody (Abcam, Cambridge, UK), anti-γH2A.X antibody (Abcam, Cambridge, UK) and propidium iodide (500 nM) (ThermoFisher Scientific, Somerset, NJ) for 4 hours. Later, the sections were washed using PBS-T and incubated with anti-rabbit Alexa flour-647 for 2 hours in the dark. Later, the sections were imaged on incubated with Hoechst 33342 (Abcam, Cambridge, UK). The sections were mounted on slides using fluorescence mounting medium and observed on a fluorescent microscope.

### Microscopy

An inverted motorized fluorescent microscope (Olympus IX81, NY) with an Orca-R2 Hamamatsu CCD camera (Hamamatsu, Japan) was used for image acquisition. Camera drive and acquisition were performed using a MetaMorph Imaging System (Olympus, NY) and Fluo View Viewer 4.2 software were used for image acquisition.

### Statistical analysis

Data are represented as means +/− standard error of mean (SEM). Student's *t*-test was performed to assess if experimental groups were significantly different from each other. All statistical analyses and graphs were generated using GraphPad Prism software.

## CONCLUSIONS

These results demonstrate the potential of Pep-1L as a delivery platform against the tumor restricted IL13RA2. Furthermore, we demonstrated the use of PET/CT to evaluate tumor targeting after CED, which can be used to both ensure that the CED catheter is properly placed and to map successful targeting by putative IL13RA2 targeted therapies. We also demonstrated the safety of arming the Pep-1L platform with Ac-225, a powerful α-particle emitter. We found significant anti-tumor effects of [^225^Ac]Pep-1L in these initial experiments, which we are continuing to investigate in GBM and other cancer models that express IL13RA2.
